# *Propionibacterium acnes* induces cartilaginous endplate degeneration by promoting MIF expression via the NF-κB pathway

**DOI:** 10.1186/s13018-020-01714-6

**Published:** 2020-06-09

**Authors:** Ying Zhang, Yuting Wang, Yanyan Yuan, Yeting Lin, Binbin Lin, Haiyan Zhou

**Affiliations:** 1 Department of Anesthesiology, Sir Run Run Shaw Hospital, Zhejiang University School of Medicine, Hangzhou, 310016 China; 2 Department of Anesthesiology, Ningbo Yinzhou No. 2 Hospital, Ningbo, 315100 China

**Keywords:** Cartilaginous endplate degeneration, Macrophage migration inhibitory factor, *Propionibacterium acnes*, NF-κB pathway, Inflammatory cytokines

## Abstract

**Background:**

*Propionibacterium acnes* (*P. acnes*) is a novel pathogenic factor that contributes to cartilaginous endplate (CEP) degeneration. However, the underlying mechanism of *P. acnes*-induced CEP degeneration remains unclear. The objective of this study is to investigate the underlying mechanism of *P. acnes*-induced CEP degeneration.

**Methods:**

We first examined MIF expression in degenerated human CEP samples by immunohistochemistry. We developed a *P. acnes*-induced rat model and detected MIF expression using immunohistochemistry. Additionally, we investigated the mechanism of *P. acnes*-induced CEP degeneration in CEP cells using western blotting and reverse transcription-quantitative polymerase chain reaction (RT-qPCR).

**Results:**

We found that compared with the normal human CEP, the expression of MIF was increased in the degenerated human CEP. In a rat model, *P. acnes* induced CEP degeneration and upregulated MIF expression significantly. More importantly, we revealed the underlying mechanism of *P. acnes*-induced CEP degeneration in the rat CEP cells. Firstly, *P. acnes* induced the expression of MIF in a concentration-dependent manner. Then, MIF upregulated the expression of MMP-13 and promoted the secretion of IL-6 and IL-1β. Finally, *P. acnes* may promote MIF expression via NF-κB pathway rather than ERK1/2 pathway.

**Conclusion:**

*P. acnes*-induced MIF expression via NF-κB pathway may be the underlying mechanism of CEP degeneration.

## Background

Intervertebral disc degeneration (IVDD) refers to the structural disruption and composition change of the intervertebral disc that can induce many common discogenic diseases, including spinal instability, disc herniation, and lower back pain [1]. These diseases not only affect people’s life and work but also significantly increase the medical burden [2, 3]. However, the etiologies of IVDD are complex, and its pathogenesis is not fully understood.

The etiologies of IVDD mainly include excessive mechanical loading, nutritional disorders and trauma factors [1]. However, since Stirling isolated *Propionibacterium acnes* (*P. acnes*) from patients with IVDD, an increasing number of more studies has focused on the effect of bacterial infection on IVDD [4]. Some studies have shown that the prevalence of *P. acnes* ranges from 13 to 44% in patients with IVDD [5]. To further verify that *P. acnes* is the cause of IVDD, our previous study found that the inoculation of *P. acnes* induced significant IVDD in rabbit models [6]. Therefore, *P. acnes* may be important in the pathogenesis of IVDD, and the underlying mechanism of *P. acnes*-induced IVDD is worth further study.

The cartilaginous endplate (CEP) is a layer of hyaline interface between the disc and vertebral bodies [7]. In a normal disc, the nutrition of the nucleus pulposus and inner layers of the annulus fibrosus depends mainly on the diffusion of nutrients through the CEP [8]. Modic changes are imaging manifestations of CEP degeneration and may be associated with degenerative disc disease and low back pain [9]. Moreover, the biological characteristics of early CEP degeneration are extracellular matrix (ECM) degradation and release of matrix metalloproteinases (MMPs) [10]. Changes in type II collagen (Col II) and matrix metalloproteinase-13 (MMP-13) are two of the most representative features of CEP degeneration [11]. Therefore, CEP degeneration is considered the initiating factor of IVDD, and inhibition of early CEP degeneration is very important to delay IVDD.

Macrophage inhibition factor (MIF) is associated with the pathogenesis of many inflammatory diseases [12]. A recent study demonstrated that MIF is expressed in degenerated human CEP and induced CEP degeneration by activating its receptor [13]. Additionally, MIF could contribute to the upregulated mRNA expression of MMP-1 and MMP-3, which are thought to be responsible for ECM degradation in rheumatoid arthritis [14]. However, few studies have focused on the initiating events that may contribute to MIF secretion. *P. acnes* is considered a novel etiology of IVDD and stimulates many cell types to produce inflammatory factors [4]. Therefore, whether *P. acnes* could induce IVDD by promoting MIF expression is worth further study.

In this study, we first sought to determine whether *P. acnes* is responsible for CEP degeneration by promoting MIF expression in vivo and in vitro. Additionally, whether activation of the NF-κB pathway is critical for MIF expression induced by *P. acnes* is worth further study. Based on our information, this study is the first to explore the relationship between *P. acnes* infection and MIF. Our findings will provide new insights to prevent and treat IVDD.

## Materials and methods

### Human CEPs

Fifteen patients participated in this study from September 2018 to March 2019. The control group (*N* = 5; male/female = 3/2; age = 23.80 ± 3.83 years) was obtained from lumbar vertebral burst fracture patients who showed no degenerative change on MRI (Pfirrmann grade I or II). The degenerated group (*N* = 10; male/female = 6/4; age = 61 ± 7.13 years) was obtained from patients with low back pain who had Modic changes on MRI (Pfirrmann grade III or IV). All tissues were dissected and fixed in 4% buffered paraformaldehyde for 48 h at 4 °C and then were submerged in 10% EDTA for 1 month for decalcification. The study was approved by the Ethical Review Board of Sir Run Run Shaw Hospital. All subjects provided written informed consent in accordance with the Declaration of Helsinki.

### Inoculation of *P. acnes* into lumbar IVDs of rats

Ten 8-week-old male Sprague-Dawley rats were used in this study. Before surgery, intraperitoneal injection of pentobarbital sodium was performed to anesthetize the rats. The discs (L3/4 to L4/5) were then punctured with a 28-gauge needle. The rats (*N* = 5 per group) were injected with 5 μl of phosphate-buffered saline (PBS) or 5 μl of *P. acnes* (ATCC 6919 provided by Guangzhou Type Culture Collection at 1.6 × 107 CFU/ml, Guangdong, China). The rats were scanned by MRI at 2 weeks, 1 month, 1.5 months, and 2 months to observe changes in the disc endplate. After 2 months, the CEP tissues were harvested and fixed in 4% buffered paraformaldehyde for 48 h at 4 °C. All animals were supplied by the Animal Center, Zhejiang University School of Medicine (Zhejiang, China) and in accordance with the Ministry of Science and Technology of the People’s Republic of China Animal Care guidelines. The animal experiments were conducted with approval from the Ethical Review Board of Sir Run Run Shaw Hospital.

### Cocultures of CEPs and *P. acnes*

CEP tissues were obtained and cultured from 8-week-old male Sprague-Dawley rats. All animals were approved by the Ethical Review Board of Sir Run Run Shaw Hospital. CEP tissues were isolated by carefully dissecting the annulus fibrous tissues, bone fragments and nucleus pulposus [15]. CEP cells were obtained according to our previous experimental procedures [16]. Briefly, the CEP tissues excised from rat IVDs were rinsed in PBS and were then cut into pieces as quickly as possible to avoid any deterioration. CEP cells were isolated by sequential digestion with type II collagenase (0.2 mg/ml) for 2 h at 37 °C incubator. The supernatant was harvested and centrifuged at 800 rpm for 5 min. The cellular pellet was resuspended in Dulbecco’s modified Eagle’s medium (DMEM, GNM12800) with 10% fetal bovine serum (FBS, Gibco, America) and was cultured in a 37 °C, 5%CO_2_ incubator. Electron microscope revealed that the cells were polygonal, the nuclei were round or oval, the nuclear membrane was obvious, and there were many unbounded membrane vacuoles under the cell membrane and in the cytoplasm. CEP cells within three generations were used to perform the experiments.

For coculture, the monoclonal standard *P. acnes* was cultured in broth for 14 days, and then the supernatant was obtained by centrifugation at 5000 rpm for 10 min, filtered with a 0.22-μm filter and stored at 4 °C. The *P. acnes* supernatant or recombinant rat MIF (rMIF) (orb168826, Biorbyt, UK) was added to the cell culture (5 × 105 cells/well) in a 6-well culture plate. After 24 or 48 h, the cocultured cells were used for subsequent experiments.

### Immunohistochemistry (IHC)

The human and rat CEP tissues were cut into 4-μm-thick sections for Safranin O-fast green, hematoxylin and eosin (H&E) staining and IHC staining as previously described [17]. Sections were stained with H&E for cell density and morphology and with safranin O fast green for proteoglycans and matrix degeneration. The immunoreactivities of MIF, MMP13, and Col II were analyzed using an SP Rabbit & Mouse HRP Κit (CW2069, CWBIO, China). Rabbit anti-MIF (ab34712, Abcam), MMP-13 (A11755, ABclonal), and Col II (ab34712, Abcam) polyclonal immunoglobulin G antibodies were used at a dilution of 1:200. The sections were imaged using a Nikon ECLIPSE 80i microscope (Nikon, Tokyo, Japan). Three pathologists, who were blinded to the group, were responsible for counting the numbers of CEP cells under high-power fields (magnification × 200) for three sections in each specimen (*N* = 3 samples/group). The integrated optical density (IOD) was analyzed by Image Pro Plus 6.0.

### Reverse transcription-quantitative polymerase chain reaction (RT-qPCR)

CEP cells were cocultured with *P. acnes* supernatant (2.5% or 5%) for 24 h. CEP cells were cultured with unstimulated or 5% *P. acnes* supernatant alone or 5% *P. acnes* supernatant and 20 μM 4-IPP (MIF inhibitor) for 24 h. They were lysed in TRIzol (Invitrogen Inc, Carlsbad, CA, USA) and total RNA was extracted using an Ultrapure RNA Κit (CW0581, CWBIO, China). Complementary DNA was synthesized using PrimeScript RT MasterMix (Takara Bio, Otsu, Japan). The qPCR was completed using the SYBR Green qPCR MasterMix (Takara Bio, Otsu, Japan). The primer sequences are shown in Table 1. Amplification was performed at 95 °C for 10 min (preincubation), 95 °C for 15 s and 60 °C for 60 s for 40 cycles (amplification), 95 °C for 15 s and 60 °C for 60 s (melting curves) and 40 °C for 5 min (cooling). Whole RT-qPCR reactions were performed in duplicate, and the amplification signals from target genes were normalized by β-actin in the same reaction. The relative mRNA levels were calculated as *x* = 2^−∆∆Ct, in which ∆∆Ct = ∆Ct E−∆Ct C, ∆Ct E = Ct exp-Ctβ-actin, and ∆Ct C = Ct C−Ct β-actin.

**Table 1:** Sequences of primers

Rat gene		Primer sequences (5′-3′)
MIF	Forward	CTTGGGTCACACCGCACTTA
	Reverse	GAGAGAAACCCCTCTGGCAC
MMP-13	Forward	CCTGGAGCCCTGATGTTTC
	Reverse	TGGGTCACACTTCTCTGGTG
Col-II	Forward	AAGGGACACCGAGGTTTCACTGG
	Reverse	GGGCCTGTTTCTCCTGAGCGT
β-actin	Forward	TATCCTGGCCTCACTGTCCA
	Reverse	AAGGGTGTAAAACGCAGCTC

### Western blotting

CEP cells were cocultured with *P. acnes* supernatant or rMIF for 48 h. CEP cells were cultured with unstimulated or 5% *P. acnes* supernatant alone or 5% *P. acnes* supernatant and 20 μM 4-IPP (MIF inhibitor) for 24 hours. They were extracted using RIPA lysis buffer (Solarbio, Beijing, China) containing a protease-inhibitor cocktail. The supernatants were collected after centrifugation at 12,000 rpm for 15 min. Proteins were separated on 10% SDS-PAGE gels and were transferred by electroblotting to PVDF membranes (Hercules, CA). The membranes were blocked (1 h) in 5% (w/v) nonfat dry milk in Tris-buffered saline containing Tween (TBST). The membranes were incubated (4 °C, overnight) with primary antibodies for MIF, MMP-13, Col II, IL-6, IL-1β, β-actin, P65, p-P65, ERΚ1/2, and p-ERΚ1/2 (1:1000 dilution, Abcam) and then were incubated with the secondary antibody horseradish peroxidase-conjugated goat anti-rabbit immunoglobulin G (1:5000 dilution; Abcam). Immunoreactive bands were detected using an electrochemical luminescence reagent (Millipore, Billerica, MA, USA) and were visualized using Image Lab software (Bio-Red, Hercules, CA, USA).

### Statistical analysis

The data were collected from three independent experiments, analyzed using SPSS version 19.0 (SPSS, Chicago, USA) and expressed as means ±S.D. Two-sided Student’s *t* test was used to analyze differences between two groups. *p* < 0.05 was considered significantly different.

## Results

### Changes in degenerated human CEPs

**Fig. 1:**
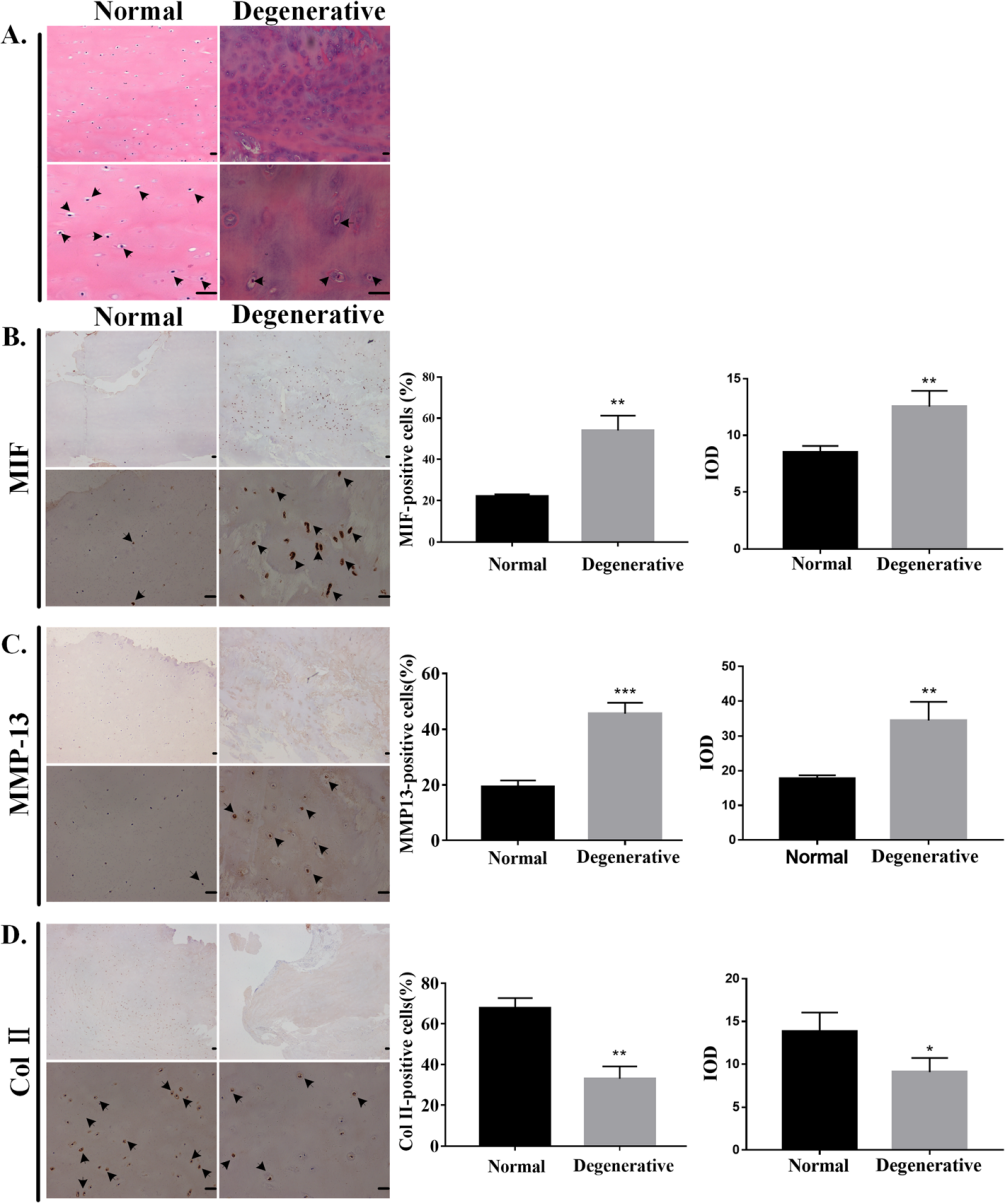
Changes in degenerated human CEPs. **a** Hematoxylin and eosin (H&E) staining is shown in the normal control and degenerated groups. Immunohistochemistry of **b** MIF, **c** MMP-13, and **d** Col II are shown in the normal control and degenerated groups. The left side is the control group and the right side is the experimental group. The upper panels were amplified at × 40, while the lower panels were amplified at × 200. The black arrow represents immunopositive cells. The quantified results are shown on the right. CEP = cartilaginous endplate. MIF = macrophage migration inhibitory factor. MMP-13 = matrix metalloproteinase-13. Col II = type II collagen. Scale bar: 20 μm. *p < 0.05, **p < 0.01, ***p < 0.001

In human CEP, H&E staining showed that cells were round and small in the normal control group, while the degenerated group showed fewer and disorderly cells (Fig. 1a). Furthermore, the expression levels of MIF (Fig. 1b) and MMP-13 (Fig. 1c) were increased in the degenerated group, while the expression of Col II (Fig. 1d) was higher in the normal control group. These results prove that MIF is sharply increased in human degenerated CEP, indicating that increased MIF expression might accelerate human CEP degeneration.

### Changes in *P. acnes*-inoculated rat CEPs

**Fig. 2:**
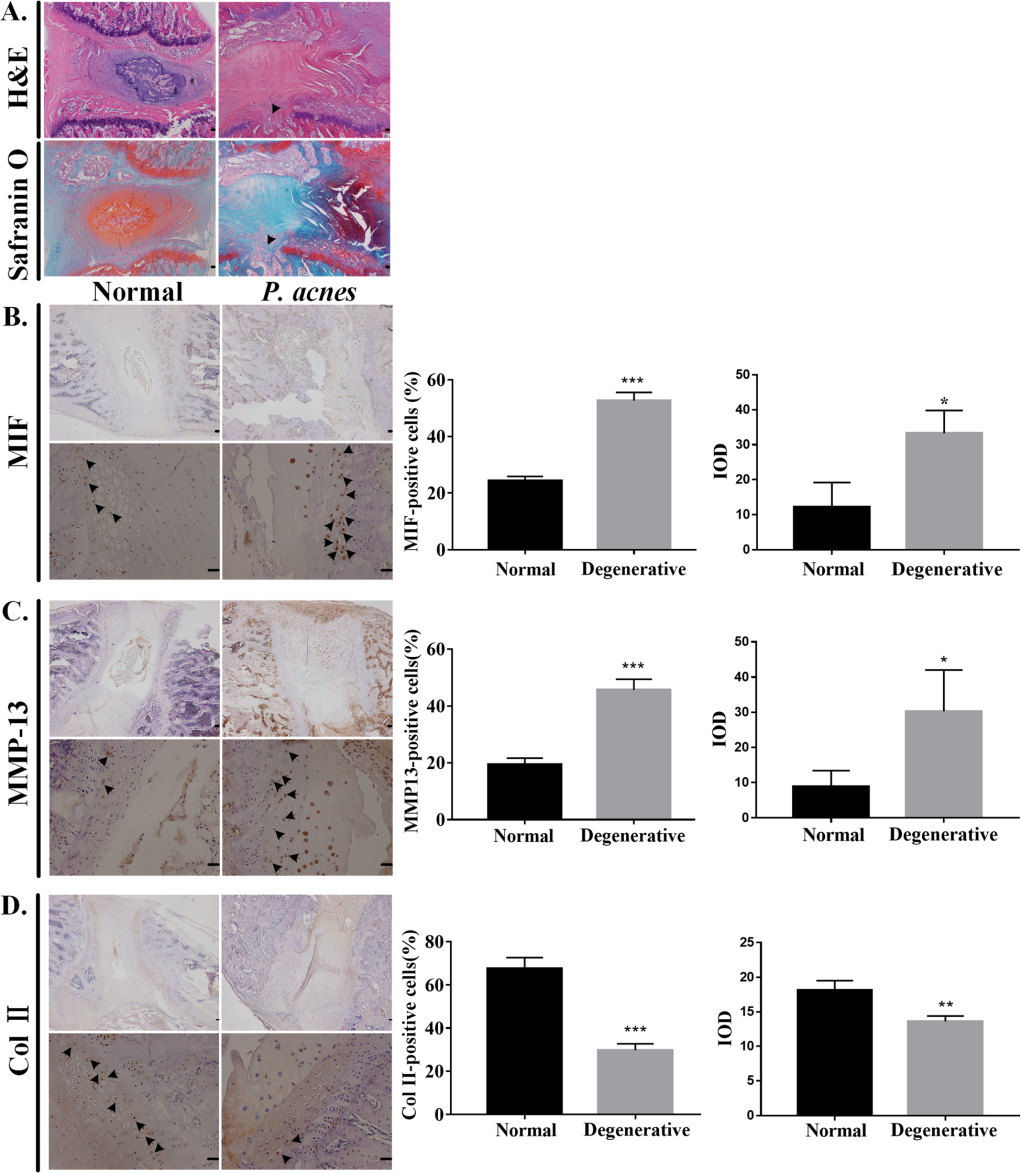
Changes in *P. acnes*-inoculated rat CEPs. **a** H&E staining and Safranin O fast green staining of the segment of *P. acnes*-inoculated intervertebral discs demonstrated the disappearance of the nucleus pulposus, endplate fracture (black arrow), and a disorganized annulus fibrosus. Immunohistochemistry of **b** MIF, **c** MMP-13, and **d** Col II showed that *P. acnes* induced CEP degeneration. The left side is the control group and the right side is the experimental group. The upper panels were amplified at × 40, while the lower panels were amplified at × 200. The black arrow represents immunopositive cells. Scale bar: 20 μm. *p < 0.05, **p < 0.01, ***p < 0.001

To elucidate the correlation between MIF and CEP degeneration by *P. acnes* infection, the bacteria were inoculated into the lumbar intervertebral discs of rats. After 2 months, H&E staining and Safranin O fast green staining showed that the *P. acnes*-inoculated segment showed endplate rupture, disappearance of the nucleus pulposus, and disorganization of the annulus fibrosus (Fig. 2a). IHC analysis showed that the expression levels of MIF (Fig. 2b) and MMP-13 (Fig. 2c) were increased significantly in *P. acnes*-inoculated CEP degeneration compared with those in the control groups, whereas the expression of Col II (Fig. 2d) was decreased significantly. Together, the results showed that *P. acnes* can induce CEP degeneration by promoting MIF expression in vivo.

### *P. acnes*-induced MIF production in CEP cells

**Fig. 3:**
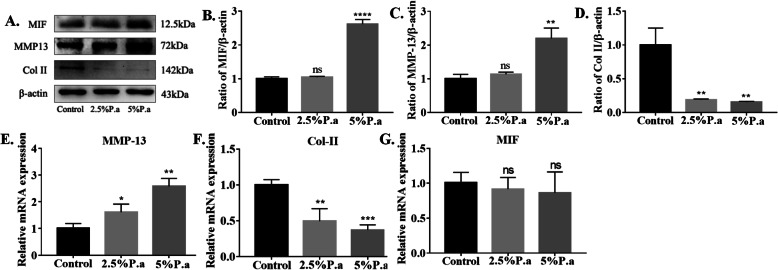
*P. acnes*-induced MIF Production in CEP cells. **a**–**d** Western blot analysis of MIF, MMP-13, and Col II expression in CEP cells infected with *P. acnes* supernatant for 48 h in a concentration-dependent manner. **e**–**f** RT-qPCR analysis of MIF, MMP-13, and Col II mRNA expression infected with *P. acnes* supernatant for 24 h. *p < 0.05, **p < 0.01, ***p < 0.001. *p* values were analyzed by two-sided Student’s *t* test. The data are expressed as means ± SD

To further demonstrate the relationship between *P. acnes* and MIF in vitro, we stimulated CEP cells with *P. acnes* supernatant (2.5% and 5%) for 24 or 48 h. First, protein analysis showed that the expression levels of MIF and MMP-13 were increased in high concentration of *P. acnes* supernatant group, while the expression of Col II was higher in the control group (Fig. 3a–d). It is worth noting that MIF gene expression (Fig. 3g) hardly changed in three groups, likely due to *P. acnes* regulating MIF at the translation stage. The gene expression levels of MMP-13 and Col II were consistent with changes in the protein levels (Fig. 3e, f).

**Fig. 4:**
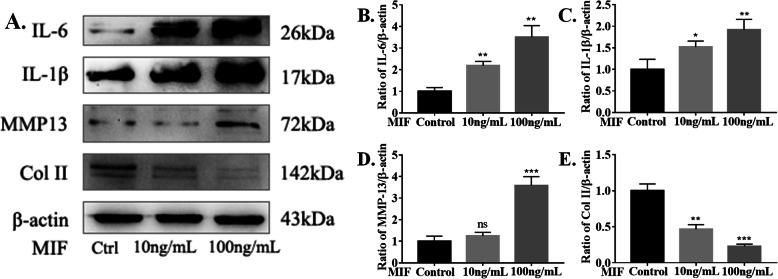
Recombinant Rat MIF (rMIF)-Induced Inflammatory Factor Production in CEP cells. **a**–**e** Western blot analysis of IL-6, IL-1β, MMP-13, and Col II expression in CEP cells infected with rMIF for 48 h in a concentration-dependent manner. *p < 0.05, **p < 0.01, ***p < 0.001. *p* values were analyzed by two-sided Student’s *t* test. The data are expressed as means ± SD

Next, to further investigate the relationship between MIF and CEP degeneration, rMIF (10 ng/ml and 100 ng/ml) was used to stimulated CEP cells for 48 h (Fig. 4a). The results showed that MIF can promote the release of inflammatory cytokines, including IL-6 and IL-1β (Fig. 4b, c). Moreover, MIF can induce CEP degeneration by regulating anabolic molecules (Col II) and catabolic molecules (MMP-13) in high concentration. (Fig. 4d, e).

Taken together, the findings show that *P. acnes* can induce CEP degeneration by activating MIF in vitro. In addition, MIF can accelerate CEP degeneration directly by itself and indirectly by releasing inflammatory factors.

**Fig. 5:**
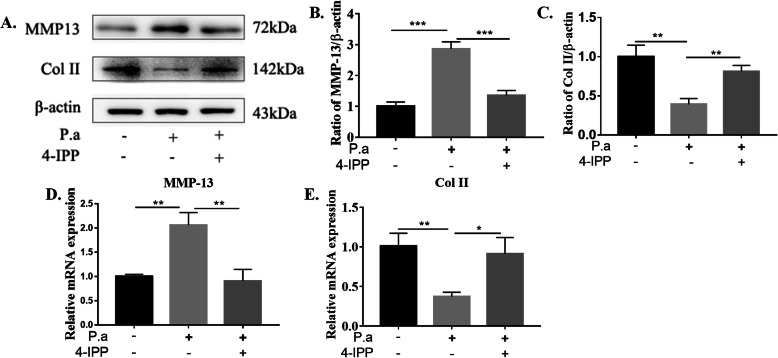
The effect of 4-IPP (MIF inhibitor) in *P. acnes*-induced CEP degeneration. **a**–**c** Western blot analysis of MMP-13 and Col II infected with 5% *P. acnes* supernatant alone or 5% *P. acnes* supernatant and 20 μM 4-IPP for 24 h. **d**–**e** RT-qPCR analysis of MMP-13 and Col II mRNA expression infected with 5% *P. acnes* supernatant alone or 5% *P. acnes* supernatant and 20 μM 4-IPP for 24 h. *p < 0.05, **p < 0.01, ***p < 0.001. *p* values were analyzed by two-sided Student’s *t* test. The data are expressed as means ± SD

More importantly, CEP cells were cultured with unstimulated or 5% *P. acnes* supernatant alone or 5% *P. acnes* supernatant and 20 μM 4-IPP (MIF inhibitor) for 24 h (Fig. 5). The results showed that blockade of MIF prevented upregulation of MMP-13 and downregulation of Col II at the protein (Fig. 5b, c) and gene levels (Fig. 5d, e). Therefore, MIF may be a novel target to prevent and treat CEP degeneration.

### *P. acnes*-induced MIF production via the NF-κB pathway

**Fig. 6:**
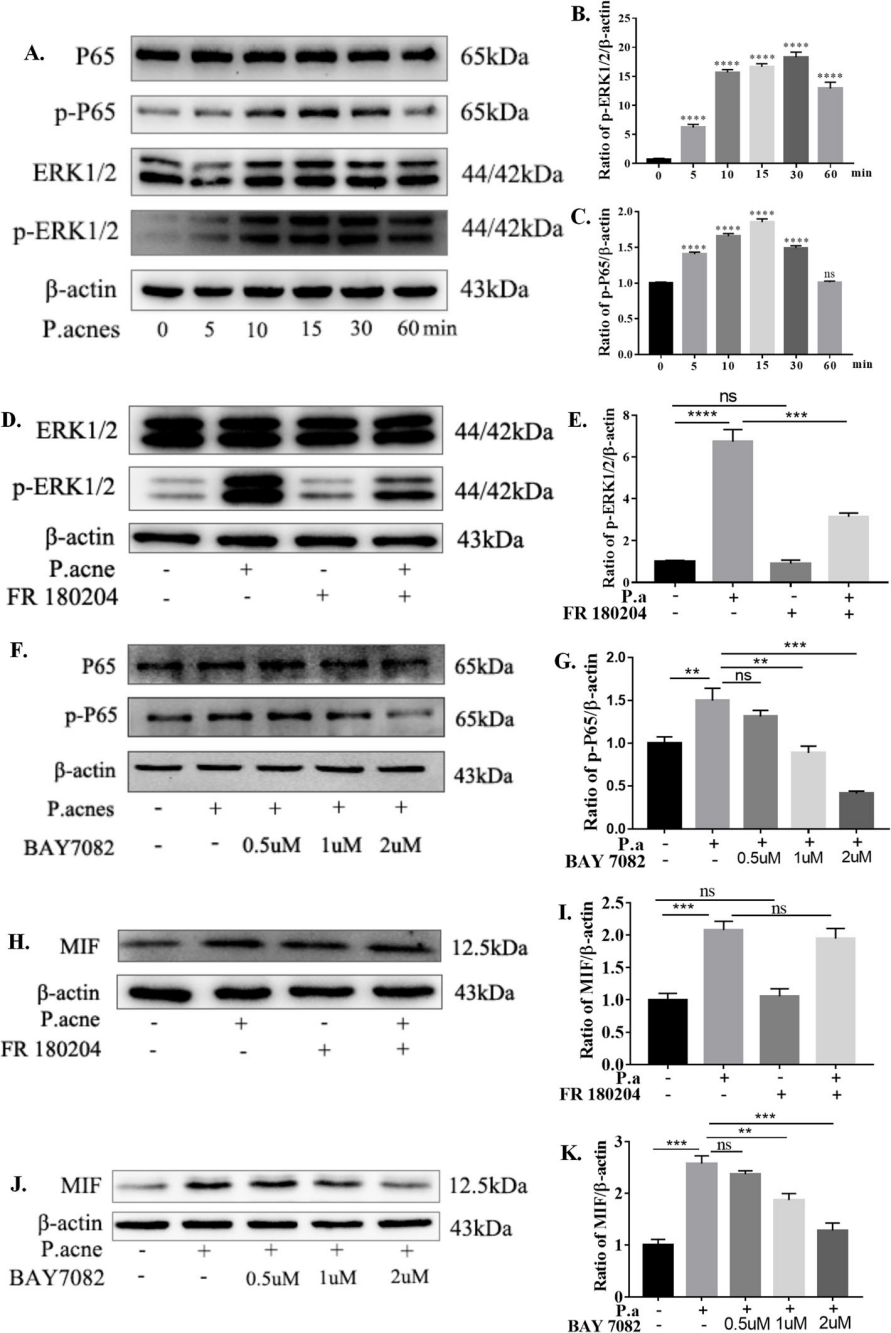
*P. acnes*-induced MIF production via the NF-κB pathway. **a***P. acnes* supernatant could activate both the NF-κB and ERΚ1/2 pathways in a time-dependent manner. **b**, **c** The ERK1/2 pathway is inhibited by FR180204, and the NF-κB pathway is inhibited by BAY-7082. **d**, **e** Western blot analysis of MIF expression infected with *P. acnes* supernatant for 48 h, pretreated with or without FR-180204 (20 μM). **f**, **g** Western blot analysis of MIF expression infected with *P. acnes* supernatant for 24 h, pretreated with or without BAY-7082 in a concentration-dependent manner. *p < 0.05, **p < 0.01, ***p < 0.001. *p* values were analyzed by two-sided Student’s *t* test. The data are expressed as means ± SD

We further explored the upstream signaling pathways of MIF expression by *P. acnes* infection. Previous reports have demonstrated that the NF-κB signaling pathway regulates *P. acnes*-induced IVDD by mediating iNOS/NO and COX-2/PGE2 expression (Lin et al. 2018). We speculated that inhibition of the NF-κB pathway could be essential to reduce the expression of MIF to prevent CEP degeneration. Additionally, ERΚ1/2 signaling was associated with the pathological mechanism of IVDD and the expression of MIF was closely related to the ERΚ1/2 pathway (Mitchell et al. 1999). However, whether activation of both pathways is critical for MIF expression induced by *P. acnes* is less clear. The results showed that *P. acnes* can activate both the NF-κB and ERΚ1/2 pathways (Fig. 6a–c), which are inhibited by FR180204 (ERK1/2 inhibitor) (Fig. 6d, e) and BAY7082 (NF-κB inhibitor) (Fig. 6f, g). However, only NF-κB pathway inhibition can decrease the expression of MIF (Fig. 6j, k); ERK1/2 pathway inhibition does not participate in this process (Fig. 6h, i). Thus, the NF-κB pathway may be involved in *P. acne*-induced MIF expression in CEP cells.

## Discussion

*P. acnes* is considered a new etiology of IVDD, and the relationship between them has gradually become a hot spot for research^4^. However, the underlying mechanism of *P. acnes*-induced IVDD remains elusive. In this study, we found that *P. acnes* induced IVDD by promoting MIF expression in human and rat CEPs. More importantly, MIF is involved in the pathogenesis of *P. acnes*-induced IVDD by regulating disc metabolism and promoting the secretion of inflammatory factors in CEP cells. Additionally, the signaling pathway involved in the expression of MIF by *P. acnes* infection was proved to be the NF-κB pathway, not the ERK1/2 pathway. These results will provide new insights to prevent and treat IVDD.

MIF is regarded as a multipotent cytokine involved in the pathogenesis of IVDD [18]. MIF is able to inhibit the synthesis of aggrecan and Col II in CEP chondrocytes, which then leads to the destruction of ECM in IVDs [19]. In addition, MIF promotes the secretion of inflammatory factors, such as IL-6, IL-8, and PGE2, in chondrocytes from human-degenerated CEPs [13]. However, few studies have focused on the initiating events that may contribute to MIF secretion in IVDD. We found that *P. acnes* induced IVDD the overexpression of MIF in vivo and in vitro. Additionally, MIF inhibition prevents upregulation of MMP-13 and downregulation of Col II in vitro. Based on our information, we first revealed the relationship between *P. acnes* and MIF. Since precious report has demonstrated that *P. acnes* induced IVDD by promoting nucleus pulposus cell apoptosis [5]. In the following study, we may detect whether the inhibition of MIF has an effect on the survival of CEP cells.

MMP-13 and Col II are regarded as the core of the ECM and are both involved in the anabolism and catabolism of intervertebral discs. In this study, we found that *P. acnes* increased the synthesis of MMP-13 and degradation of Col II in vivo and in vitro. In addition, inflammatory cytokines have been reported to exert negative regulation on the synthesis of the ECM [17, 20]. MIF was crucial for Col II degradation by inducing MMP-1 and MMP-9 secretion [21]. To further investigate the direct effect of MIF in IVDD, the different concentrations of rMIF were used to stimulate the rat CEP cells. We found the expression of Col II was significantly reduced in exogenous MIF group. Meanwhile, we investigated that MIF promoted the secretion of inflammatory mediators, such as IL-6 and IL-1β. There are two assumptions of IVDD induced by MIF: The direct effect and the indirect effect by MIF, such as the increase of IL-6 and IL-1β, on the ECM expression in CEP degeneration. However, there were no attempts to functionally validate MIF in a rat model. Further investigation about this issue should be conducted in the future.

Activation of the NF-κB signaling pathway is vital for the balance between cartilage degradation and synthesis [22]. MIF appears to regulate cellular function via downstream signaling pathways, such as NF-κB [23]. Two NF-κB binding sites have been reported to be present in the MIF proximal promoter region by promoter analysis [24]. In our previous study, we found that the reduction of MIF expression after lycorine treatment is likely due to the suppression the NF-κB signaling pathway [15]. However, whether activation of the NF-κB pathway is critical for MIF expression induced by *P. acnes* is less clear. We found that *P. acnes* could activate the NF-κB signaling pathway, and NF-κB pathway inhibition (BAY7082) significantly reduced the expression of MIF in a dose-dependent manner. In addition, ERK1/2 signaling participated in the pathological mechanism of IVDD [25]. Inhibition of ERK1/2 signaling pathway could decrease the secretion of inflammatory cytokines induced by MIF [19]. Interestingly, MIF expression was not reduced significantly with the increase of ERK1/2 inhibition. These results show that the signaling pathway involved in the expression of MIF by *P. acnes* infection was proved to be the NF-κB pathway, not the ERK1/2 pathway.

## Conclusion

The study demonstrated that *P. acnes* induces CEP degeneration by promoting MIF production, and the NF-κB signaling pathway may participate in this process. The novel mechanism reported for the first time reveals the relationship between *P. acnes* and MIF. More importantly, MIF could become a new target to prevent and treat intervertebral disc degeneration, which will receive extensive attention and research in the future.

## Data Availability

The datasets used and/or analyzed during the current study are available from the corresponding author on reasonable request.

## Abbreviations

IVDD: Intervertebral disc degeneration
*P. acnes*: *Propionibacterium acnes*
MIF: Macrophage inhibition factor
CEP: Cartilaginous endplate
ECM: Extracellular matrix
MMPs: Matrix metalloproteinases

## References

[1] Modic MT, Ross JS (2007). Lumbar Degenerative Disk Disease. Radiology Volume..

[2] Andersson GBJ. Epidemiological features of chronic low-back pain. The Lancet.1999; 354: (9178)581-585. 10.1016/s0140-6736(99)01312-4. 10.1016/S0140-6736(99)01312-4 10470716

[3] Katz BJN (2006). Lumbar Disc Disorders and Low-Back Pain: Socioeconomic Factors and Consequences. The Journal of Bone & Joint Surgery..

[4] Stirling A, Worthington T, Rafiq M, Lambert PA, Elliott TSJ (2001). Association between sciatica and Propionibacterium acnes. The Lancet..

[5] Lin Y, Tang G, Jiao Y, et al. Propionibacterium acnes Induces Intervertebral Disc Degeneration by Promoting iNOS/NO and COX-2/PGE2 Activation via the ROS-Dependent NF-kappaB Pathway. Oxid Med Cell Longev. 2018. 10.1155/2018/3692752. 10.1155/2018/3692752 PMC6120277 30210652

[6] Shan Z. P. acnes incubation in the discs results in time-dependent modic changes. SPINE.2017; Volume 42, Number 21, pp 1595–1603. 10.1097/BRS.0000000000002192 28399545

[7] Chan WC, Sze KL, Samartzis D, Leung VY, and Chan D. Structure and biology of the intervertebral disk in health and disease. Orthop Clin North Am2011; 42(4): 447-464, 10.1016/j.ocl.2011.07.012. 10.1016/j.ocl.2011.07.012 21944583

[8] Giers MB, Munter BT, Eyster KJ (2017). Biomechanical and endplate effects on nutrient transport in the intervertebral disc. World Neurosurgery..

[9] Nguyen C, Poiraudeau S, Rannou F (2012). Vertebral subchondral bone. Osteoporos Int..

[10] Kang R, Li H, Ringgaard S, et al. Interference in the endplate nutritional pathway causes intervertebral disc degeneration in an immature porcine model.International orthopaedics. 2014;38(5): 1011-7. 10.1007/s00264-014-2319-9. 10.1007/s00264-014-2319-9 PMC3997759 24652423

[11] Zhang JF, Wang GL, Zhou ZJ, Fang XQ, Chen S, and Fan SW. Expression of Matrix Metalloproteinases, Tissue Inhibitors of Metalloproteinases, and Interleukins in Vertebral Cartilage Endplate. Orthop Surg2018; 10(4): 306-311. 10.1111/os.12409. 10.1111/os.12409 PMC6594525 30474324

[12] Baugh JA, Bucala R. Macrophage migration inhibitory factor. Crit Care Med Vol. 2002;30, No. 1 (Suppl.). 11782558

[13] Xiong C, Huang B, Cun Y, Aghdasi BG, Zhou Y (2014). Migration inhibitory factor enhances inflammation via CD74 in cartilage end plates with Modic type 1 changes on MRI. Clin Orthop Relat Res..

[14] Lue H, Kleemann R, Calandra T, Roger T, Bernhagen J (2002). Macrophage migration inhibitory factor (MIF): mechanisms of action and role in disease. Microbes and Infection..

[15] Wang G, Huang K, Dong Y (2018). Lycorine Suppresses Endplate-Chondrocyte Degeneration and Prevents Intervertebral Disc Degeneration by Inhibiting NF-kappaB Signalling Pathway. Cell Physiol Biochem..

[16] Huang B, Chen J, Zhang X, et al. Alpha 2-Macroglobulin as Dual Regulator for Both Anabolism and Catabolism in the Cartilaginous Endplate of Intervertebral Disc. Spine (Phila Pa 1976)2019; 44(6): E338-E347. 10.1097/BRS.0000000000002852. 10.1097/BRS.0000000000002852 30138255

[17] Tang P, Zhu R, Ji WP (2016). The NLRP3/Caspase-1/Interleukin-1beta axis is active in human lumbar cartilaginous endplate degeneration. Clin Orthop Relat Res..

[18] Neidlinger-Wilke C, Boldt A, Brochhausen C (2014). Molecular interactions between human cartilaginous endplates and nucleus pulposus cells: a preliminary investigation. Spine.

[19] Xiong C, Huang Y, Kang H, Zhang T, Xu F, and Cai X. Macrophage inhibition factor-mediated CD74 signal modulate inflammation and matrix metabolism in the degenerated cartilage endplate chondrocytes by activating extracellular signal regulated kinase 1/2. Spine2017;42(2): E61-E70. 10.1097/BRS.0000000000001726. 10.1097/BRS.0000000000001726 27270637

[20] Kepler CK, Ponnappan RK, Tannoury CA, Risbud MV, Anderson DG (2013). The molecular basis of intervertebral disc degeneration. Spine J.

[21] Zernecke A, Bernhagen J, and Weber C. Macrophage migration inhibitory factor in cardiovasculardisease. Circulation.2008;117(12):15941602. 10.1161/CIRCULATIONAHA.107.729125. 10.1161/CIRCULATIONAHA.107.729125 18362243

[22] Zhao Y, Li Z, Wang W (2016). Naringin protects against cartilage destruction in osteoarthritis through repression of NF-kappaB signaling pathway. Inflammation.

[23] Gore Y, Starlets D, Maharshak N, et al. Macrophage migration inhibitory factor induces B cell survival by activation of a CD74-CD44 receptor complex. The Journal of Biological Chemistry.2008;283: 2784-2792.10.1074/jbc.M703265200. 10.1074/jbc.M703265200 18056708

[24] Chen L, Yang G, Zhang X (2009). Induction of MIF expression byoxidized LDL via activation of NF-kappaB in vascular smooth muscle cells. Atherosclerosis..

[25] Wei Y, Zhi-hong W, Gui-xing Q (2013). Extracellular signal regulated kinase inhibition modulates rat annulus fibrosus cell response to interleukin-1. Spine..

